# Supplementary material for: Intensity vs. Volume in Professional Soccer: Comparing Congested and Non-Congested Periods in Competitive and Training Contexts Using Worst-Case Scenarios
Source: Sports (Basel). 2025 Feb 27;13(3):70. doi: 10.3390/sports13030070 (PMC11946561; doi:10.3390/sports13030070)
Supplement: Supplementary file 1 [file sports-13-00070-s001.zip › sports-3492266-supplementary.pdf]

**Table S1.** Playing time for each player during a season.

| Player | Playing Time | % of Possible Playing Time | % of the Total Playing Time | Added Playing Time |
|---|---|---|---|---|
| Player 1 | 3811 | 100% | 9% | 9% |
| Player 2 | 3811 | 100% | 9% | 18% |
| Player 3 | 3811 | 100% | 9% | 27% |
| Player 4 | 3514 | 92% | 8% | 36% |
| Player 5 | 3437 | 90% | 8% | 44% |
| Player 6 | 3274 | 86% | 8% | 52% |
| Player 7 | 2861 | 75% | 7% | 59% |
| Player 8 | 2400 | 63% | 6% | 65% |
| Player 9 | 2131 | 56% | 5% | 70% |
| Player 10 | 2074 | 54% | 5% | 75% |
| Player 11 | 1738 | 46% | 4% | 79% |
| Player 12 | 1613 | 42% | 4% | 83% |
| Player 13 | 1498 | 39% | 4% | 86% |
| Player 14 | 1181 | 31% | 3% | 89% |
| Player 15 | 1008 | 26% | 2% | 92% |
| Player 16 | 950 | 25% | 2% | 94% |
| Player 17 | 749 | 20% | 2% | 96% |
| Player 18 | 662 | 17% | 2% | 97% |
| Player 19 | 374 | 10% | 1% | 98% |
| Player 20 | 326 | 9% | 1% | 99% |
| Player 21 | 317 | 8% | 1% | 100% |
| Player 22 | 106 | 3% | 0% | 100% |
| Player 23 | 0 | 0% | 0% | 100% |
| Player 24 | 0 | 0% | 0% | 100% |
